# Rifaximin Modifies Gut Microbiota and Attenuates Inflammation in Parkinson’s Disease: Preclinical and Clinical Studies

**DOI:** 10.3390/cells11213468

**Published:** 2022-11-02

**Authors:** Chien-Tai Hong, Lung Chan, Kai-Yun Chen, Hsun-Hua Lee, Li-Kai Huang, Yu-Chen S. H. Yang, Yun-Ru Liu, Chaur-Jong Hu

**Affiliations:** 1 Department of Neurology, Shuang Ho Hospital, Taipei Medical University, New Taipei City 23561, Taiwan; 2 Department of Neurology, School of Medicine, College of Medicine, Taipei Medical University, Taipei 11031, Taiwan; 3 Ph.D. Program in Medical Neuroscience, College of Medical Science and Technology, Taipei Medical University, Taipei 11031, Taiwan; 4 Joint Biobank, Office of Human Research, Taipei Medical University, Taipei 11031, Taiwan

**Keywords:** rifaximin, gut microbiota, Parkinson’s disease, inflammation

## Abstract

Patients with Parkinson’s disease (PD) exhibit distinct gut microbiota, which may promote gut-derived inflammation. Rifaximin is a nonabsorbable antibiotic that can modify gut microbiota. The present study investigated the effect of rifaximin on gut microbiota and inflammation status in PD. The study examined the effect of long-term rifaximin treatment on in vivo transgenic PD mice (MitoPark) and short-term rifaximin treatment on patients with PD. Rifaximin treatment caused a significant change in gut microbiota in the transgenic PD mice; in particular, it reduced the relative abundance of *Prevotellaceae UCG-001* and increased the relative abundance of *Bacteroides*, *Muribaculum*, and *Lachnospiraceae UCG-001*. Rifaximin treatment attenuated serum interleukin-1β, interleukin-6 and tumor necrosis factor-α, claudin-5 and occludin, which indicated the reduction of systemic inflammation and the protection of the blood–brain barrier integrity. The rifaximin-treated MitoPark mice exhibited better motor and memory performance than did the control mice, with lower microglial activation and increased neuronal survival in the hippocampus. In the patients with PD, 7-day rifaximin treatment caused an increase in the relative abundance of *Flavonifractor* 6 months after treatment, and the change in plasma proinflammatory cytokine levels was negatively associated with the baseline plasma interleukin-1α level. In conclusion, the present study demonstrated that rifaximin exerted a neuroprotective effect on the transgenic PD mice by modulating gut microbiota. We observed that patients with higher baseline inflammation possibly benefited from rifaximin treatment. With consideration for the tolerability and safety of rifaximin, randomized controlled trials should investigate the disease-modification effect of long-term treatment on select patients with PD.

## 1. Introduction

Parkinson’s disease (PD) is the second most common neurodegenerative disease [1]. The deposition of Lewy bodies formed by the aggregation of α-synuclein in the midbrain substantia nigra is the pathological hallmark of PD [2]. The intestines are hypothesized to be the site of origin of pathological α-synuclein, which is transmitted to the brain and then to the medulla through the vagus nerve [3]. Numerous studies have demonstrated the accumulation of α-synuclein in the colonic tissue of patients with early- and prodromal-stage PD [4,5,6]. Moreover, findings related to the gut–brain axis strongly support the association between the intestinal environment and neurodegeneration [7].

Gut microbiota (GM) are microorganisms present in the intestines; they are a crucial determinant of the intestinal environment. The GM profile affects food and drug metabolism and absorption as well as local and systemic inflammatory responses, and it is affected by systemic conditions, such as stress and depression [8]. GM is crucial for the aggregation of α-synuclein, and the absence of microorganisms in the intestines prevented the aggregation of pathological α-synuclein in transgenic PD mice [9]. The GM profile differs between patients with PD and healthy individuals [10,11,12]. Although GM profile is strongly associated with diet, the enrichment of the genera *Lactobacillus*, *Akkermansia*, and *Bifidobacterium* and the depletion of bacteria belonging to the family *Lachnospiraceae* and the genus *Faecalibacterium* are consistently noted [13].

GM can be modified by probiotics and prebiotics. Prebiotics, such as starches, provide a favorable environment for the enrichment of beneficial microorganisms. Probiotics are select microorganisms such as *Lactobacillus* and *Bifidobacterium* that can be orally prescribed and that can colonize the intestines [14]. Antibiotics have an adverse effect on GM. Systemic antibiotic treatment reduces the diversity of GM; increases the proportion of resistant bacterial strains, which induce a focal intestinal inflammatory response; disrupts the tight junction in the intestines; and causes the influx of intestinal toxins into the bloodstream (leaky gut syndrome) [15].

Rifaximin is a nonaminoglycoside, semisynthetic, nonsystemic antibiotic derived from rifamycin SV [16]. Rifaximin is approved by the United States Food and Drug Administration for the treatment of travelers’ diarrhea [17] and hepatic encephalopathy and for the prevention of bacterial outgrowth in patients with liver cirrhosis [18]. Rifaximin exhibits unique eubiotic characteristics and does not reduce either the diversity of GM or the abundance of beneficial bacterial strains [19]. Rifaximin has been used in patients with PD to prevent bacterial overgrowth in the small intestine. Treatment with 1100 mg of rifaximin per day for 7 days significantly reduced the growth of undesirable bacterial strains, especially *Helicobacter pylori*, with minimal side effects [20]. Regarding neuroprotection, rifaximin treatment reduced the serum neurofilament light chain levels in patients with mild to moderate Alzheimer disease [21].

We hypothesized that rifaximin modifies GM and exerts beneficial effects on patients with PD through the gut–brain axis. A preclinical study investigated the protective effect of long-term rifaximin treatment on transgenic PD mice. This phase I/IIa open-label clinical study investigated the effect of 7-day rifaximin treatment on GM and systemic inflammatory responses in patients with PD.

## 2. Methods

### 2.1. Study Participants and Rifaximin Treatment

Twenty participants were enrolled in this study. PD was diagnosed on the basis of the United Kingdom Parkinson’s Disease Society Brain Bank Diagnostic Criteria. Only patients with mild to moderate PD, defined as stage I to III PD according to the Hoehn and Yahr scale, were included in the PD group. This study was approved by the Joint Institutional Review Board of Taipei Medical University (approval no. N201005044) and registered at ClinicalTrials.gov (Identifier: NCT03958708). In this open-label, single-arm study, all the patients with PD were prescribed 550 mg of rifaximin twice per day for 7 days. Baseline GM, clinical performance, and blood samples were examined before rifaximin treatment. GM was assessed immediately and 6 months after treatment, whereas motor performance and blood samples were examined 6 months after treatment. Side effects of rifaximin were recorded immediately and 6 months after treatment. For the clinical study, rifaximin was provided by CK Medtech Corporation, Taiwan.

### 2.2. Experimental Animals and Rifaximin Treatment

All animal procedures were approved and performed in accordance with the guidelines of the Institutional Animal Care and Use Committee/Laboratory Animal Center of Taipei Medical University (LAC-2020-0308). Transgenic *DAT-cre × Tfam^loxP^* MitoPark mice [22] were maintained in the animal unit of Taipei Medical University in accordance with Taiwan’s regulations for experimental animals. The mice were maintained in a temperature-controlled room (22 °C) under a 12-hour light–dark cycle. Transgenic MitoPark mice were provided by the National Laboratory Animal Center, Taiwan. At the age of 8 weeks, the mice were divided into two groups and fed either 50 mg/kg rifaximin 5 days per week or continual normal diet for up to 3 months. In total, 6 MitoPark mice were treated with rifaximin, and another 6 MitoPark mice were not. One MitoPark mouse in the non-treated group was dead during the experimental period. In order to mimic the clinical study (PD patients only), the in vivo study did not include wide-type mouse. Regarding the in vivo study, rifaximin was purchased from Sigma-Aldrich Inc. (St. Louis, MO, USA).

### 2.3. GM: 16S rRNA Assessment

DNA was extracted from stool samples (approximately 200 mg) by using the QIAamp Fast DNA Stool Mini Kit (Catalog no. 51604; Qiagen, Germantown, MD, USA) according to the manufacturer’s instructions. The amount of buffer was adjusted in proportion to the amount of stool. Subsequently, 16S ribosomal RNA (rRNA) gene amplification and library construction were performed following protocols provided by Illumina [https://support.illumina.com/downloads/16s_metagenomic_sequencing_library_preparation.html (assessed on 22 February 2022)]. The V3–V4 region of bacterial 16S rRNA genes was amplified through polymerase chain reaction by using the universal bacterial primers 341F (5′-CCTACGGGNGGCWGCAG-3′) and 805R (5′-GACTACHVGGGTATCTAATCC-3′), which contain Illumina overhang adapter sequences in the forward (5′-TCGTCGGCAGCGTCAGATGTGTATAAGAGACAG-3′) and reverse (5′-GTCTCGTGGGCTCGGAGATGTGTATAAGAGACAG-3′) primers. Illumina sequencing adapters and dual-index barcodes were added to amplicon targets by using the Nextera XT Index kit. The quantity and quality of libraries were measured using the QSep100 Analyzer (BiOptic, New Taipei City, Taiwan). Finally, the libraries were normalized and pooled in an equimolar ratio and sequenced using an Illumina MiSeq System.

The 16S rRNA gene sequencing data were analyzed as follows. The universal primers were removed from demultiplexed paired reads by using Cutadapt [version 1.12; https://github.com/marcelm/cutadapt (assessed on 22 February 2022)]. Subsequently, sequences were processed using the DADA2 workflow [version 1.6; http://bioconductor.org/packages/dada2/ (assessed on 22 February 2022)] in the R environment. In brief, filtering, trimming, and dereplication were independently performed on the forward and reverse reads, and a denoising algorithm was then applied to the reads. The paired reads were merged, and they required a minimum 20 bp overlap. Chimeras were subsequently removed. The taxonomy of the inferred ribosomal sequence variants (SVs) was determined using the SILVA database [version 138; http://www.arb-silva.de (assessed on 22 February 2022)] as a reference, with the minimum bootstrap confidence being 80. Multiple sequence alignment of the SVs was performed using DECIPHER [version 2.6.0; http://bioconductor.org/packages/DECIPHER/ (assessed on 22 February 2022)], and a phylogenetic tree was constructed from the alignment using phangorn [version 2.3.1; https://cran.r-project.org/package=phangorn (assessed on 22 February 2022)]. A phyloseq object was created using information on the frequency table, taxonomy assignment, and phylogenetic tree, and community analyses were performed using phyloseq [version 1.19.1; http://bioconductor.org/packages/phyloseq/ (assessed on 22 February 2022)]. Raw abundances were converted into normalized abundances by using the getVarianceStabilizedData function of DESeq2 [version 1.18.1; http://bioconductor.org/packages/DESeq2/ (assessed on 22 February 2022)] after the phyloseq data were converted into a DESeq2 object by using the phyloseq_to_deseq2 function. The exact Wilcoxon rank-sum test (Mann–Whitney U test) was performed to determine differentially abundant taxonomic ranks between the control and rifaximin-treated mice and between the control and rifaximin-treated participants with PD at different time points. We analyzed sample relatedness by calculating UniFrac distances by using the GUniFrac package (version 1.1) to determine the community dissimilarity between the groups. Principal coordinate analysis (PCoA) ordination of UniFrac distances was performed, and the adonis and betadisper functions in the vegan package [version 2.4; https://CRAN.R-project.org/package=vegan (assessed on 22 February 2022)] were used to statistically analyze the dissimilarity of compositions among the groups and the homogeneity of dispersion, respectively.

### 2.4. Protein Quantification: Western Blot Analysis

Total proteins were extracted from frozen midbrain, cortical, and hippocampal tissue samples after homogenization in ice-cold RIPA buffer (Millipore, Temecula, CA, USA) containing protease (Sigma-Aldrich Inc., St. Louis, MO, USA) and a phosphatase inhibitor (BioShop, Ontarrio, Canada). Protein concentrations were measured using the Bradford protein assay (Bio-Rad, Hercules, CA, USA). In total, 20 μg of protein was separated in a 4–20% sodium dodecyl sulfate–polyacrylamide gradient gel (TOOLS, New Taipei City, Taiwan). After electrophoresis, the proteins were transferred onto Hybond-enhanced chemiluminescence nitrocellulose membranes. Antibodies against interleukin (IL)-1β (Cell Signaling Technology, Beverly, MA, USA, Cat.#12242, 1:1000), IL-6 (Cell Signaling Technology, Cat. #12912, 1:1000), tumor necrosis factor (TNF)-α (Cell Signaling Technology, Cat.# 11948, 1:1000), claudin-5 (Abcam, Burlingame, CA, USA, ab131259, 1:1000), occludin (Abcam, ab216327, 1:1000), CD-86 (Cell Signaling Technology, Cat. #91882, 1:1000) and arginase-1 (Cell Signaling Technology, Cat. #43933, 1:1000) were used. β-Actin (Millipore, MAB1501, 1:10,000) was used as a loading control in the mouse tissue. For the mouse and human plasma, equal loading was achieved using identical volumes of samples.

### 2.5. Immunohistochemistry

Brain tissues were collected from the MitoPark mice and perfused with 4% paraformaldehyde. The tissues were incubated at 4 °C for postfixation overnight. The tissues were cut into 5 μm slices. Parafilm slides were deparaffinized using Trilogy (Cell Marque Corporation, Hot Springs, AR, USA) at 120 °C for 15 min and washed with tap water several times. The slides were blocked with phosphate-buffered saline containing 0.5% Triton X-100 (*v*/*v*) and 5% bovine serum albumin. Anti-tyrosine hydroxylase (TH) (Millipore, AB152, 1:1000), anti-NeuN (Millipore, Burlington, MA, USA, MAB377, 1:100), and anti-ionized calcium-binding adapter molecule 1 (Iba1) (Genetex, Irvine, CA, USA, GTX100042, 1:100) antibodies were used. The slides were incubated with the primary antibody overnight. After washing, a peroxidase-labeled polymer conjugated to a secondary antibody (Agilent, Santa Clara, CA, USA) was added, and the slides were incubated for 30 min. Subsequently, the slides were stained with a Dako REAL EnVision Detection system (Agilent) at room temperature for 5 min. Images were obtained using the MoticEasyScan Pro 6 Imaging System (Motic, Schertz, TX, USA). Microglia morphology analysis was performed using ImageJ [23] according to previous literature [24]. In brief, the obtained Iba1 immunohistochemistry images over substania nigra and hippocampus went through the protocols of skeleton analysis. The number of branches and the length of branches were obtained and averaged.

### 2.6. Human Plasma Cytokine Quantification

Six biomarkers (IL-1α, IL-1β, IL-6, IL-10, interferon (IFN)-γ, and TNFα) in the plasma samples of the participants with PD were concurrently measured using the Multiplex enzyme-linked immunosorbent assay (ELISA) kit for human cytokine panel 1 (6-Plex; MEK1010, BOSTER, Pleasanton, CA, USA). A 5-point calibration curve was prepared using undiluted and 1:9-, 1:27-, 1:81-, and 1:729-diluted aliquots of the reconstituted calibrator in the aforementioned kit through serial dilution and loaded along with blank and undiluted samples in duplicate onto the assay plate. The assay was performed following the manufacturer’s instructions.

### 2.7. Clinical Assessments

The baseline demographic data of all the participants were obtained through interviews. The cognitive function of study participants was evaluated by trained nurses by using the Taiwanese versions of the Mini-Mental Status Examination (MMSE) and Montreal Cognitive Assessment (MoCA). All the study participants were evaluated using Part I, II, and III of the Unified Parkinson’s Disease Rating Scale (UPDRS) during outpatient visits. The time between the most recent dose of anti-PD medication and the assessment of UPDRS Part IV was not recorded; the patients with PD were assumed to be on their “on” time.

### 2.8. Behavioral Assessments

The motor balance and coordination of the mice were evaluated using the beam walking and rotarod tests, respectively. For the beam walking test, the MitoPark mice were trained for 3 days to walk along a narrow Plexiglas beam (100 cm long, 0.5 cm wide) toward a home cage located at one end of the beam. The mean time required to walk across the beam was used as a measure of motor coordination. For the rotarod test, a rotating rod (Rotarod, Ugo Basile, Washington, DC, USA) was used to evaluate the motor coordination and balance of the mice. The accelerating protocol was started at a speed of 5 revolutions per minute (rpm) and reached 40 rpm within 300 s. The time to fall was the primary endpoint. For the MitoPark mice, both tests were performed five times at 30 min intervals monthly.

The novel object recognition (NOR) test procedure was reported in our previous study. In brief, the MitoPark mice were administered the NOR test in a black plastic box (45 × 45 × 60 cm^3^) with a camera at the top. The mice were habituated and trained before testing. Each mouse was placed in the box for 15 min to freely explore the environment on days 1 to 3. On day 3, each mouse was trained for 15 min in the box by using two identical objects, and they were then returned to their cages to wait for 4 h. The mouse was then placed back into the box with one familiar object and one novel object, and a video was recorded for 15 min. Objects and the test box were cleaned with 70% ethanol before each test. The video was analyzed using Noldus software (Noldus, Leesburg, VA, USA). The NOR index indicated the percentage of total time spent on the novel object and was calculated as follows: NOR = [(time of novel object)/(time of novel object + time of familiar object)] × 100). Three independent experiments were performed monthly.

Gait was analyzed during spontaneous walking by using an automated gait analysis system (Gaitlab, Viewpoint, France). Images of the mice were captured from below by using a high-speed camera (B150 frames per second) while they ran along a narrow glass corridor (790 cm) to identify paw step positions and moving speed. Different metrics were calculated, namely speed, stride length, stance time, swing time, and number of strides per second.

## 3. Results

### 3.1. Rifaximin Treatment Altered the GM of Transgenic PD Mice

**Figure 1:**
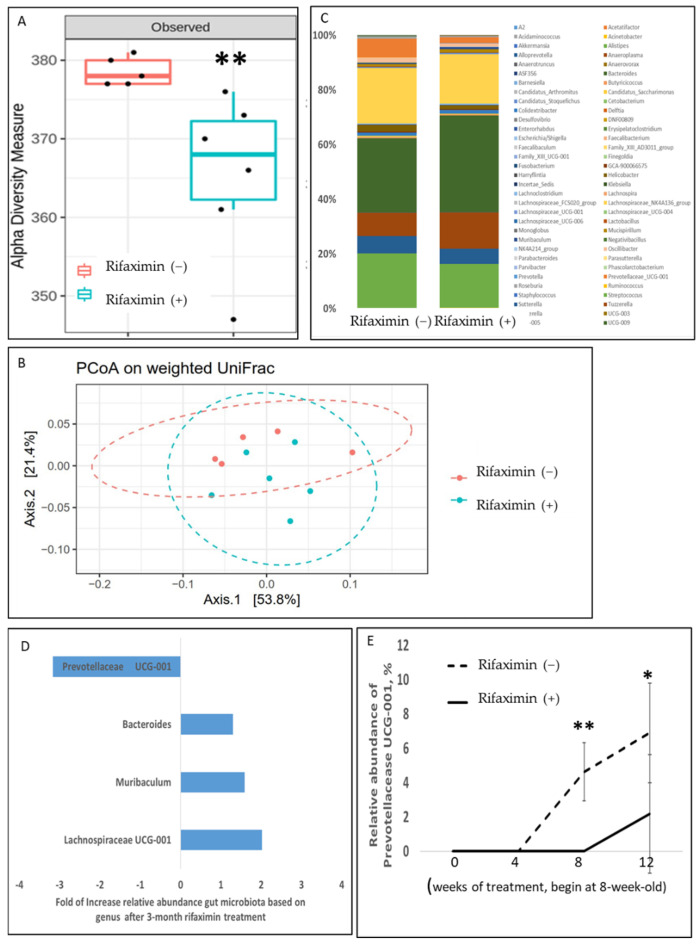
Effect of rifaximin treatment on the alteration of gut microbiota in MitoPark mice. The stool samples of the MitoPark mice that were or were not treated with rifaximin were prepared for fecal microbiome profiling through the high-throughput sequencing of the 16S rRNA gene by using the Illumina MiSeq system. (**A**) Alpha diversity of the rifaximin-treated samples and nontreated controls after 3-month treatment. (**B**) A weighted principal coordinate analysis (PCoA) plot based on the UniFrac distances of the rifaximin-treated samples and nontreated controls after 3-month treatment. (**C**) The taxonomic analysis of GM revealed the relative abundance of bacterial genera in the MitoPark mice that were or were not treated with rifaximin over 3 months. (**D**) Differences in the relative abundance of bacteria genera between the MitoPark mice that were or were not treated with rifaximin. (**E**) Changes in the relative abundance of *Prevotellaceae UCG-001* in the MitoPark mice that were or were not treated with rifaximin during experiments. Data are presented as mean ± standard deviation. *, *p* < 0.05, **, *p* < 0.01.

Continuous rifaximin treatment for 3 months significantly altered the GM of the transgenic PD mice (MioPark). The GM alpha diversity of the rifaximin-treated MitoPark mice significantly differed from that of the control MitoPark mice (*p* = 0.008) (Figure 1A). The results of weighted UniFrac PCoA revealed that the fecal microbiome profile significantly differed between the rifaximin-treated MitoPark mice and nontreated MitoPark mice (*p* = 0.02) (Figure 1B). A taxonomic analysis of GM demonstrated a significant reduction in the relative abundance of the genera *Prevotellaceae UCG-001* and an increase in *Bacteroides*, *Muribaculum*, and *Lachnospiraceae UCG-001* after 3 months of rifaximin treatment in the MitoPark mice (Figure 1C,D). *Prevotellaceae UCG-001* was not detectable at the beginning of the experiments in both mouse groups (in 8-week-old mice). The relative abundance increased with age in the nontreated MitoPark mice but not in the rifaximin-treated MitoPark mice (Figure 1E).

### 3.2. Rifaximin Treatment Preserved Intestinal Epithelial Integrity and Reduced Systemic Inflammation in Transgenic PD Mice

**Figure 2:**
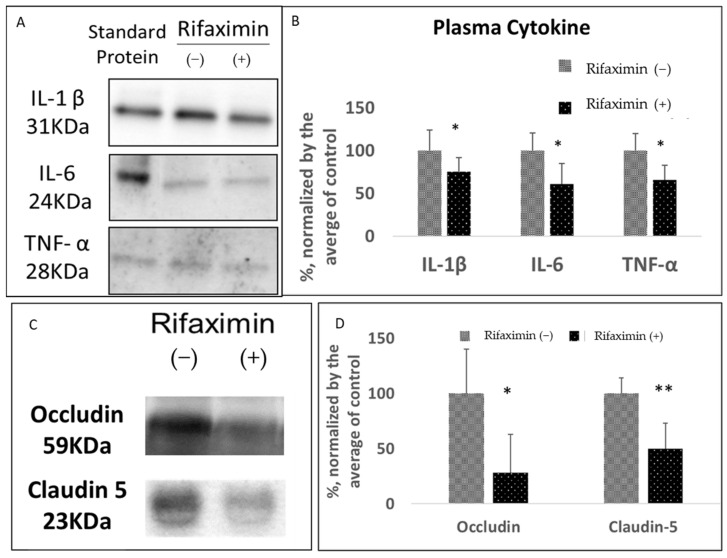
Effect of rifaximin treatment on the serum markers of inflammation and the blood–brain barrier in MitoPark mice. The serum samples of the MitoPark mice that were or were not treated with rifaximin for 3 months were obtained after the end of experiments before sacrifice. (**A**,**B**) The representative image and densitometry analysis results related to serum proinflammatory cytokines, namely IL-1β, IL-6, and TNF-α, in the MitoPark mice that were or were not treated with rifaximin for 3 months. Standard IL-1β (10 pg/mL), IL-6 (2 ng/mL), and TNF-α (0.5 ng/mL) were as the reference. (**C**,**D**) Representative image and densitometry analysis results related to the serum markers of blood–brain barrier permeabilization, namely claudin-5 and occludin, in the MitoPark mice that were or were not treated with rifaximin for 3 months. Data are presented as mean ± standard deviation. *, *p* < 0.05, **, *p* < 0.01.

Gut dysbiosis causes inflammation in the intestines and affects intestinal epithelial integrity (also known as leaky gut syndrome), which increases systemic inflammation. Systemic inflammation results in the permeabilization of the blood–brain barrier (BBB) [25]. The present study investigated whether rifaximin treatment attenuates these detrimental effects. Rifaximin treatment significantly reduced the serum concentrations of proinflammatory cytokines IL-1β, IL-6, and TNF-α in the MitoPark mice (representative image, Figure 2A and analysis, Figure 2B, Supplementary Figure S1 for the full blot). Furthermore, the rifaximin-treated MitoPark mice had a significant reduction in blood claudin-5 and occludin, which are markers of BBB permeabilization [26,27] (representative image in Figure 2C and analysis results in Figure 2D). These results suggested that the robust anti-inflammatory effect of rifaximin prevented leaky gut syndrome-associated systemic inflammation and subsequent BBB permeabilization.

### 3.3. Rifaximin Treatment Prevented Motor and Cognitive Dysfunction in Transgenic PD Mice

**Figure 3:**
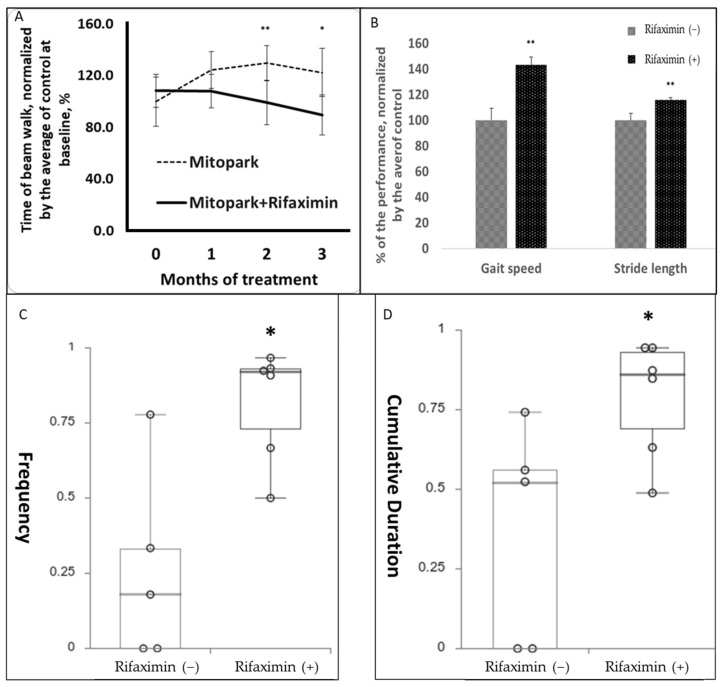
Effect of rifaximin treatment on motor and memory function in MitoPark mice. The behavioral test results of MitoPark mice that were or were not treated with rifaximin for 3 months. (**A**) Rifaximin treatment prevented a decline in the motor performance of the MitoPark mice in the beam walk assessment after 2 and 3 months of treatment. (**B**) The MitoPark mice that were treated with rifaximin for 3 months exhibited higher gait speed and longer stride length than did the nontreated MitoPark mice. (**C**,**D**) The dot and box plot demonstrated that the rifaximin-treated MitoPark mice were significantly more likely to make contact with the novel object (frequency) and stay longer (cumulative duration) with the novel object in the novel object recognition assessment, which indicated better memory. Data are presented as the mean ± standard deviation (**A**,**B**) or medians with the first and third quartiles (**C**,**D**). *, *p* < 0.05, **, *p* < 0.01.

The rifaximin-treated MitoPark mice exhibited significantly better motor activity in the beam walk test than did the nontreated MitoPark mice. The rifaximin-treated MitoPark mice required a shorter time to complete the beam walk test after 2 and 3 months of treatment compared with the control MitoPark mice that exhibited gradual deterioration (Figure 3A). Gait analysis performed 3 months after rifaximin treatment revealed a significant increase in gait speed (43.2% faster) and stride length in the rifaximin-treated MitoPark mice (15.6% longer; Figure 3B). To investigate whether rifaximin can ameliorate the memory decline in the MitoPark mice, we compared the NOR index of the MitoPark mice that received and did not receive rifaximin treatment. The results revealed significant increases in the NOR index from 52% (no treatment) to 81% (rifaximin treatment; *p* < 0.05 (Figure 3C)), and the cumulative length of time spent near the novel object (control, 59%; rifaximin, 78%; *p* < 0.05 (Figure 3D)).

### 3.4. Rifaximin Treatment Attenuated Neuroinflammation in the Midbrain and Neurodegeneration in the Hippocampus of Transgenic PD Mice

**Figure 4:**
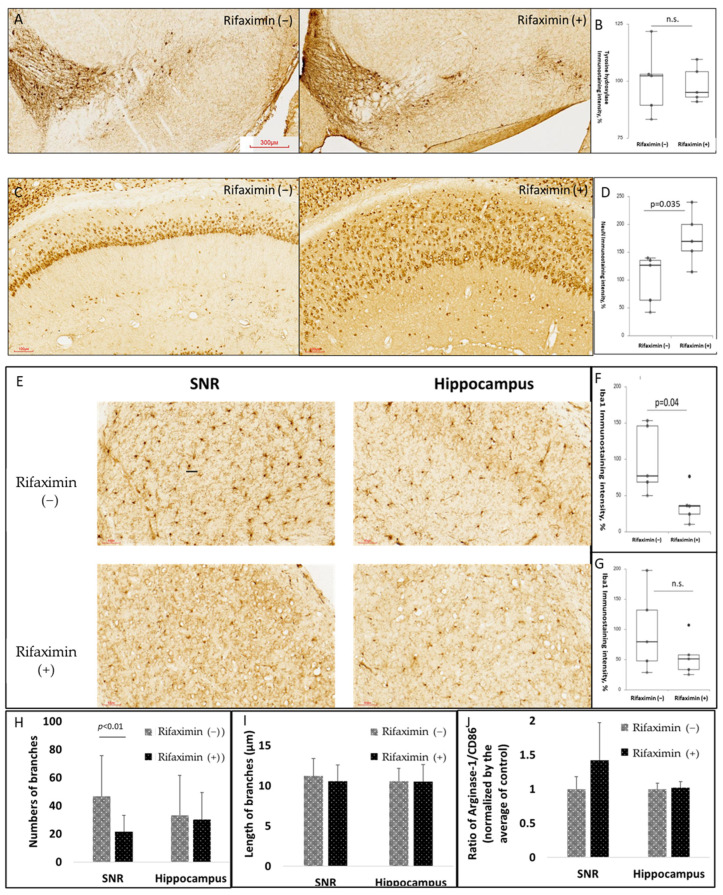
Effect of rifaximin treatment on neuroprotection and neuroinflammation in MitoPark mice. The quantification of neuronal survival, microglial activation, and proinflammatory cytokines in the brains of MitoPark mice. (**A**,**B**) Representative immunohistochemistry (IHC) images of tyrosine hydroxylase (TH), dopaminergic neuron markers, and immunoreactivity in the midbrain substantia nigra (SNR) of the MitoPark mice that were or were not treated with rifaximin. (**C**,**D**) Representative IHC images of neuronal nuclear protein (NeuN), neuronal markers, and immunoreactivity in the hippocampus of the MitoPark mice that were or were not treated with rifaximin. (**E**–**G**) Representative IHC images of ionized calcium-binding adapter molecule 1 (Iba1), microglia markers, and immunoreactivity in the midbrain substantia nigra and hippocampus of the MitoPark mice that were or were not treated with rifaximin. (**H**,**I**) The morphological analysis of the stained microglia by the numbers of branches and the length of the branches. (**J**) The ratio between M2 microglia to M1 microglia, defined by the expression level of arginase-1 and CD86, was analyzed in the midbrain SNR and hippocampus. Data are presented as mean ± standard deviation. n.s., non-significant.

To determine the neuroprotective effect of rifaximin on PD, we examined the neuronal viability of the MitoPark mice. We observed that rifaximin treatment did not prevent the loss of midbrain dopaminergic neurons in the MitoPark mice (Figure 4A,B). However, rifaximin treatment significantly preserved neuronal viability in the hippocampus (Figure 4C,D). Furthermore, considering the anti-inflammatory property of rifaximin, we examined the activation of microglia. The rifaximin-treated MitoPark mice had a significant reduction in Iba1 immunostaining in the midbrain substantia nigra (SNR). Moreover, a similar but nonsignificant reduction in Iba1 immunostaining was noted in the hippocampus of the MitoPark mice (Figure 4E–G). The morphology of microglia, assessed by the number of branches (Figure 4H) and the length of the branches (Figure 4I) in the midbrain SNR and hippocampus, were also analyzed. It was found that the number of branches significantly reduced in the midbrain SNR, without a significant difference in the length of the branches. In general, the deramification (reduced the branches) of the microglia indicated the activation of the microglia, no matter the activation of M1 (pro-inflammatory) or M2 (anti-inflammatory) microglia. We further investigated the ratio of the expression of arginase-1 (a marker of M2 microglia) to CD86 (a marker of M1 microglia) in the midbrain SNR and hippocampus. We found a trend of increased M2 microglia in the midbrain SNR (Figure 4J for densitometry analysis and Supplementary Figure S2 for representative images).

### 3.5. Effect of Rifaximin Treatment on Patients with PD: Alterations in GM

**Figure 5:**
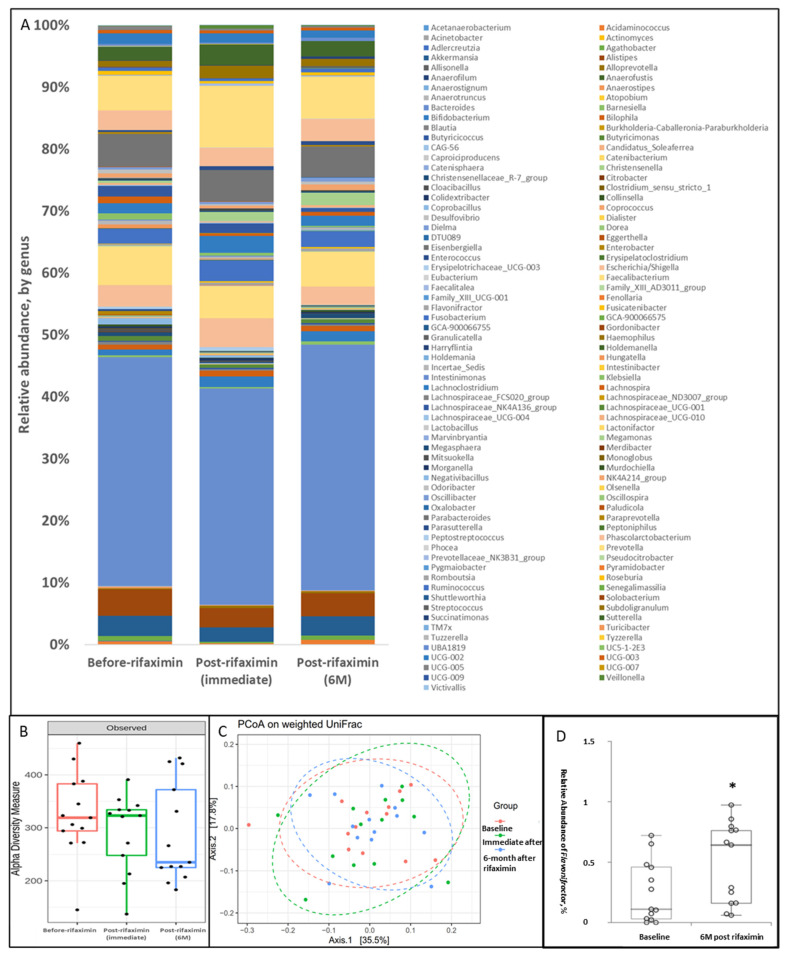
Effect of 7-day rifaximin on patients with PD: Alteration in GM. The stool samples of the study participants before, immediately, and 6 months after rifaximin treatment were prepared for fecal microbiome profiling through the high-throughput sequencing of the 16S rRNA gene by using the Illumina MiSeq system. (**A**) The taxonomic analysis of GM revealed the relative abundance of bacterial genera in the study participants at three time points. (**B**) Alpha diversity of the GM in the study participants at three time points. (**C**) A weighted principal coordinate analysis (PCoA) plot based on UniFrac distances for the study participants at three time points. (**D**) The difference in the relative abundance of *Flavonifractor* gena in the study participants before and 6 months after 7-day rifaximin treatment. Data are presented as medians with the first and third quartiles, *, *p* < 0.05.

This open-label, single-arm clinical trial involving the administration of 1100 mg of rifaximin treatment per day for 7 days was conducted at Taipei Medical University-Shuang Ho Hospital. The first enrollment was conducted in June 2018, and the last one was in March 2020. In total, 20 patients with early to middle stage PD signed informed consent forms. Two patients withdrew from the study before the initiation of treatment, one patient failed to collect their stool sample before the prescription of rifaximin, and one patient was excluded due to a change in diagnosis from idiopathic PD to progressive supranuclear palsy during the follow-up period. The stool samples of three other study participants were not suitable for the analysis due to contamination, incorrect storage, or being of poor quality. Finally, 13 study participants, six female, with mean age of 61.59 ± 5.34 years as well as 1.77 ± 1.74 years of disease duration completed biological sample collection (stool samples before, immediately after, and 6 months after rifaximin treatment and blood samples before and 6 months after rifaximin treatment) and clinical assessment (UPDRS part III before and 6 months after rifaximin treatment) (demographic data shown in Table 1). Rifaximin did not change the overall relative abundance (Figure 5A), α-diversity (*p* = 0.20) (Figure 5B), and β-diversity (*p* = 0.94) (Figure 5C) of GM immediately and 6 months after 1-week rifaximin treatment. Rifaximin treatment resulted in a significant increase in the relative abundance of *Flavonifractor* (Figure 5D) but no other bacterial genera in the patients with PD (Supplementary Figure S3). No severe side effect or adverse event was reported.

### 3.6. Effect of Rifaximin Treatment on Patients with PD: Serum Cytokine Profile

**Figure 6:**
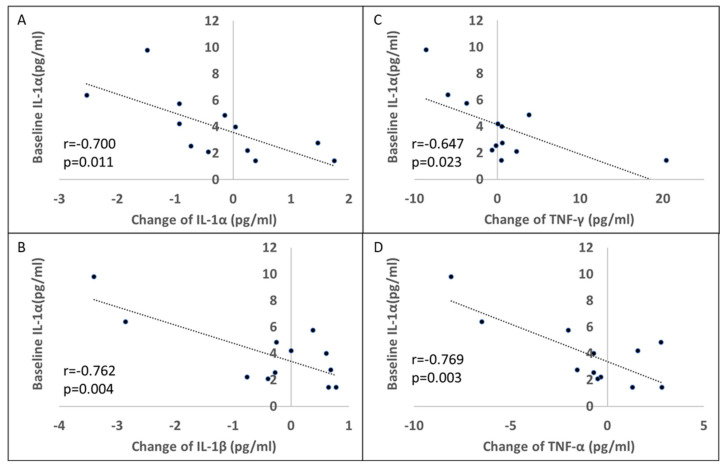
Effect of 7-day rifaximin on patients with PD: Association with baseline interleukin (IL)-1α. The association between the baseline plasma IL-1α level and changes in plasma IL-1α (**A**), IL-1β (**B**), IFN-γ (**C**), and TNF-α (**D**) levels before and 6 months after 7-day rifaximin treatment.

One-week rifaximin treatment caused an increased trend of serum anti-inflammatory cytokine, IL-10 increase (baseline:15.88 ± 8.60, 6-month post-rifaximin: 26.82 ± 25.32 pg/mL, *p* = 0.06) without significant change in the rest of the serum cytokine profile of the patients with PD (Table 2). However, a significant negative correlation was observed between the baseline IL-1α level and changes in the levels of the proinflammatory cytokines IL-1α, IL-1β, IFN-γ, and TNF-α (Figure 6A–D). These results indicated that the anti-inflammatory effect of rifaximin treatment may be more robust in patients with a higher baseline inflammatory status.

## 4. Discussion

The present study investigated the effect of rifaximin on PD experimentally and clinically. Rifaximin treatment significantly altered GM in the transgenic PD mice (MitoPark); it reduced *Prevotellaceae UCG-001* levels and increased *Bacteroides, Muribaculum*, and *Lachnospiraceae UCG-001* levels. Moreover, rifaximin treatment reduced systemic inflammatory responses, protected the BBB’s epithelial function, prevented motor dysfunction and cognitive impairment, and alleviated neuroinflammation in the MitoPark mice. In the patients with PD, 7-day rifaximin treatment increased the abundance of *Flavonifractor* 6 months after treatment without causing a significant change in plasma cytokine levels. However, changes in proinflammatory cytokine levels were negatively associated with baseline IL-1α levels. These results indicated that long-term rifaximin treatment exerted a neuroprotective effect on the MitoPark mice by modifying GM and attenuating inflammation. Thus, short-term rifaximin treatment may not be adequate to exert a prolonged effect; however, patients with higher baseline inflammation were more likely to experience a reduction in systemic inflammation following 7-day rifaximin treatment. This is the first study to investigate the neuroprotective and anti-inflammatory effects of rifaximin on PD in vivo and in clinical settings. The findings of the in vivo model indicated that select patients with PD and hyperinflammation can benefit from rifaximin treatment.

The mutual link between the gut and brain has been identified in recent years [28,29]. GM is a major determinant of the gut environment. GM is essential for digestion and prevents the entry of pathogens into the body. In addition, GM is essential for the synthesis of several key components, including neurotransmitters, hormones, and fatty acids [30]. Neurological diseases affect the composition of GM, and gut dysbiosis in turn contributes to neurodegeneration [31]. GM markedly differs between patients with PD and healthy controls [10,32,33,34]. However, the causal relationship between the differences in GM and PD remains unclear. Although *Lactobacillus* and *Bifidobacterium* are typically recognized as beneficial bacterial strains, their abundances increase in patients with PD. By contrast, the family *Lachnospiraceae* and the genus *Faecalibacterium* are believed to be pathogenic GM, and their numbers are reduced in patients with PD [13]. Rifaximin modulates GM in humans by increasing the abundance of *Lactobacilli* and *Eubacteriaceae* and reducing the abundance of *Roseburia*, *Haemophilus*, *Veilonella*, and *Streptococcus* [35,36,37]. Additionally, rifaximin treatment protects gut permeability and reduces plasma lipopolysaccharide levels [38,39]. In the present study, the results demonstrated that continuous rifaximin treatment modified GM in the transgenic PD mice. Several abundant genera were significantly altered after treatment. *Prevotellaceae UCG-001* was not detected in the 8- and 12-week-old nontreated MitoPark mice, and the relative abundance of this genus increased with age, indicating the progression of neurodegeneration. Rifaximin treatment delayed this age-associated increase and significantly attenuated the relative abundance of *Prevotellaceae UCG-001* in the 16- and 20-week-old mice. The abundance of *Prevotella* was associated with inflammatory disorders. Additionally, the increased abundance of *Prevotella* has been associated with persistent inflammation in the gut and subsequent mucosal dysfunction and systemic inflammation [40,41]. Furthermore, *Prevotellaceae* is associated with periodontal disease [42] and inflammatory bowel diseases [43]. *Provotellaceae* breaks down the mucosal barrier by their secretion of sulfatases. The interaction of *Provotellaceae* with the inflammasome accounts for their pro-inflammatory role [44]. Reducing the abundance surge of *Provotellaceae UCG-001* by rifaximin may explain the benefit of rifaximin on anti-inflammation.

In addition to *Prevotellaceae*, rifaximin changed the abundance levels of other major bacterial genera in the gut of the MitoPark mice; rifaximin caused a significant increase in the relative abundance of *Bacteriodes*, *Muribaculum*, and *Lachnospiraceae UCG-001*. *Bacteroides*, the most abundant genus in the gut, can exert both beneficial and detrimental effects; however, *Bacteriodes* are essential for health because they reinforce the epithelial barrier and ameliorate inflammation by producing anti-inflammatory molecules such as polysaccharide A, sphingolipids, and outer membrane vesicles [45,46]. *Muribaculum* and *Lachnospiraceae* produce short-chain fatty acids as metabolites with anti-inflammatory properties in the gut and are associated with longevity [47,48]. The findings of this study indicated that alterations in GM caused by rifaximin treatment reduced inflammation and blood proinflammatory cytokine levels, preserved intestinal tight junction integrity, and prevented BBB breakdown. However, in our clinical study, short-term rifaximin treatment did not modify GM 6 months after treatment, and significant alteration was noted only in the relative abundance of *Flavonifractor*. The relative abundance of *Flavonifractor* is associated with the severity of PD motor symptoms and the disease stage [49,50]. The increase in the relative abundance of *Flavonifractor* in the present study may be due to a long disease duration among the study participants.

Systemic inflammation is a crucial factor in the pathogenesis of PD [51]. The association between inflammatory bowel disease and PD risk indicates the contribution of systemic inflammation to neurodegeneration [52,53]. Nonsteroidal anti-inflammatory drugs, especially ibuprofen, have been demonstrated to reduce the risk of PD [54,55]. Gut dysbiosis and subsequent leaky gut syndrome result in the entry of pathogens and toxins into systemic blood flow, thus triggering gut-derived inflammation. Furthermore, systemic inflammation leads to the impairment of the BBB, thus inducing the infiltration of immune cells into the brain and activating microglia-dependent neuroinflammation [25,56]. Studies have reported the presence of elevated proinflammatory cytokine levels in the serum and plasma extracellular vesicles of patients with PD [57,58,59]. In the present study, rifaximin treatment significantly altered GM and downregulated blood proinflammatory cytokine levels. It has been studied that the invasion of intestinal bacteria into systemic blood circulation is responsible for the dysfunction of BBB. Low-dose antibiotic treatment in the early life of experimental animals upregulated the expression of tight junction proteins of BBB [60]. Furthermore, bacterial products, such as pilli and cell wall components, trigger the pro-inflammatory cytokines and damage the BBB function [61]. The benefit of rifaximin, which reduced systemic inflammation, may contribute to the prevention of BBB breakdown, which was evaluated by examining blood claudin-5 and occludin levels.

A reduction in microglial activation and proinflammatory cytokine levels was observed in the midbrain and cortex of the rifaximin-treated MitoPark mice. Rifaximin exerts protective effects possibly through the gut–blood–brain inflammatory response axis. Because of considerable variations in baseline inflammatory status, no significant difference in plasma cytokine levels was noted before and after treatment in the patients with PD. However, a significant negative association was observed between the baseline IL-1α level and changes in the levels of other proinflammatory cytokines, including IL-1α, IL-1β, IFN-γ, and TNF-α, before and 6 months after 7-day rifaximin treatment. An elevated systemic inflammatory response is known to predict the progression of PD. Baseline C-reactive protein levels (≥0.7 mg/L) were significantly associated with greater motor decline [62]. In addition, it was found that an inflammatory summary score, including five inflammatory markers, at baseline predicted cognitive decline in patients with PD [63]. The present study may suggest that rifaximin treatment is more likely to attenuate inflammation in patients with PD with higher baseline systemic inflammation, who are at risk of progression.

The present study demonstrated that in the transgenic PD mice, rifaximin treatment altered GM and attenuated systemic inflammation, which may result in the preservation of BBB integrity, a reduction in neuroinflammation, and protection against neuronal loss. Moreover, rifaximin treatment prevented the deterioration of motor and memory functions in the transgenic PD mice. Given that rifaximin is a 99% nonabsorbable antibiotic [64], its anti-inflammatory and neuroprotective effects most likely resulted from the gut, especially through the modification of GM. This study demonstrated the role of the gut–brain axis in the pathogenesis of PD and the significance of gut-derived inflammation in PD. Although short-term rifaximin treatment did not exert a significant beneficial effect on patients with PD, the results indicated that rifaximin treatment may be beneficial for patients with baseline hyperinflammation. The safety of long-term rifaximin treatment has been confirmed in patients with hepatic encephalopathy who continuously received rifaximin to prevent intestinal bacterial outgrowth [65]. The present study has some limitations. In the transgenic PD mice, neuroprotection in midbrain dopaminergic neurons was not noted despite the preservation of motor function. MitoPark mouse models are established through the dopaminergic neuron-specific inactivation of mitochondrial transcription factor A [22]. The detrimental effect of mitochondrial dysfunction may overwhelm the modulation of neuroinflammation for neuroprotection. The preservation of motor function may result from better cognition or other nondopaminergic neuron-dependent movements, such as serotonergic neurons in the brainstem, which are responsible for the gait [66,67]. Multiple factors, including inflammation, are involved in the pathogenesis of PD. Prescription of rifaximin may not universally benefit all patients with PD, and patients with gut dysbiosis and a proinflammatory status may be suitable candidates for further clinical trials. Lastly, the detected serum cytokines of MitoPark mice were the precursors but not the cleaved, active form, which was short, half-lived, and degraded rapidly in serum.

## 5. Conclusions

The present study demonstrated that rifaximin exerted a neuroprotective effect on transgenic PD mice by modulating GM and the gut–brain axis. The clinical study suggested that patients with gut dysbiosis and a proinflammatory status would benefit from rifaximin treatment. With consideration for the tolerability and safety of rifaximin, randomized controlled trials should investigate the disease modification effect of long-term rifaximin treatment on select patients with PD.

## Figures and Tables

**Table 1:** Demographic data of study participants. The demographic data of 13 PD patients who were analyzed. Data are presented as mean± standard deviation for continuous variables.

	*n* = 13
Female	6
Age (year-old)	61.59 ± 5.34
Disease duration (years)	1.77 ± 1.74
UPDRS part III	
Baseline	13.69 ± 8.75
6-month post rifaximin	12.31 ± 9.21
MMSE	27.92 ± 2.33

UPDRS, Unified Parkinson’s Disease Rating Scale; MMSE, mini-mental status examination.

**Table 2:** Serum Cytokine profile. The change in serum cytokine level between baseline and 6-month post-rifaximin treatment in PD patients. Data are presented as mean ± standard deviation (pg/mL).

	Baseline	6-Month Post-Rifaximin	*p* Value
Interleukin-1α	4.83 ± 3.94	4.25 ± 2.70	0.21
Interleukin-1β	17.26 ± 2.88	16.88 ± 1.96	0.32
Interleukin-6	4.42 ± 1.24	4.52 ± 1.77	0.80
Interleukin-10	15.88 ± 8.60	26.82 ± 25.32	0.06
Interferon-γ	7.73 ± 4.41	8.41 ± 5.39	0.72
Tumor necrosis factor-α	13.96 ± 8.59	13.13 ± 7.47	0.37

## Data Availability

Please contact the corresponding author (C.-T.H.). The availability of data and materials requires permission from TMU-JIRB.

## Supplementary Materials

The following supporting information can be downloaded at: https://www.mdpi.com/article/10.3390/cells11213468/s1, Figure S1: The full blot of MitoPark serum cytokine analysis; Figure S2: The representative western blot analysis for the assessment of the ratio of the expression of arginase-1 (a marker of M2 microglia) to CD86 (a marker of M1 microglia) in the midbrain substantia nigra (SNR) and hippocampus; Figure S3: The difference in the relative abundance of major bacterial gena in the study participants before and 6 months after 7-day rifaximin treatment.

## Abbreviations

BBB	blood-brain barrier
ELISA	enzyme-linked immunosorbent assay
GM	gut microbiota
IFN	interferon
IL	interleukin
Iba1	ionized calcium-binding adapter molecule 1
MMSE	Mini-Mental Status Examination
NOR	novel object recognition
PD	Parkinson’s disease
PCoA	principal coordinate analysis
rRNA	ribosomal RNA
rpm	revolutions per minute
SV	sequence variants
TH	tyrosine hydroxylase
TNF	tumor necrosis factor
UPDRS	Unified Parkinson’s Disease Rating Scale

## References

[1] de Lau L.M., Breteler M.M. (2006). Epidemiology of Parkinson’s disease. Lancet Neurol..

[2] Spillantini M.G., Schmidt M.L., Lee V.M.Y., Trojanowski J.Q., Jakes R., Goedert M. (1997). α-Synuclein in Lewy bodies. Nature.

[3] Kim S., Kwon S.-H., Kam T.-I., Panicker N., Karuppagounder S.S., Lee S., Lee J.H., Kim W.R., Kook M., Foss C.A. (2019). Transneuronal Propagation of Pathologic α-Synuclein from the Gut to the Brain Models Parkinson’s Disease. Neuron.

[4] Shannon K.M., Keshavarzian A., Dodiya H.B., Jakate S., Kordower J.H. (2012). Is alpha-synuclein in the colon a biomarker for premotor Parkinson’s disease? Evidence from 3 cases. Mov. Disord..

[5] Shannon K.M., Keshavarzian A., Mutlu E., Dodiya H.B., Daian D., Jaglin J.A., Kordower J.H. (2012). Alpha-synuclein in colonic submucosa in early untreated Parkinson’s disease. Mov. Disord..

[6] Hilton D., Stephens M., Kirk L., Edwards P., Potter R., Zajicek J., Broughton E., Hagan H., Carroll C. (2014). Accumulation of α-synuclein in the bowel of patients in the pre-clinical phase of Parkinson’s disease. Acta Neuropathol..

[7] Klingelhoefer L., Reichmann H. (2015). Pathogenesis of Parkinson disease—The gut–brain axis and environmental factors. Nat. Rev. Neurol..

[8] Fan Y., Pedersen O. (2021). Gut microbiota in human metabolic health and disease. Nat. Rev. Microbiol..

[9] Sampson T.R., Debelius J.W., Thron T., Janssen S., Shastri G.G., Ilhan Z.E., Challis C., Schretter C.E., Rocha S., Gradinaru V. (2016). Gut Microbiota Regulate Motor Deficits and Neuroinflammation in a Model of Parkinson’s Disease. Cell.

[10] Lin C.H., Chen C.C., Chiang H.L., Liou J.M., Chang C.M., Lu T.P., Chuang E.Y., Tai Y.C., Cheng C., Lin H.Y. (2019). Altered gut microbiota and inflammatory cytokine responses in patients with Parkinson’s disease. J. Neuroinflammation.

[11] Aho V.T.E., Pereira P.A.B., Voutilainen S., Paulin L., Pekkonen E., Auvinen P., Scheperjans F. (2019). Gut microbiota in Parkinson’s disease: Temporal stability and relations to disease progression. EBioMedicine.

[12] Hopfner F., Künstner A., Müller S.H., Künzel S., Zeuner K.E., Margraf N.G., Deuschl G., Baines J.F., Kuhlenbäumer G. (2017). Gut microbiota in Parkinson disease in a northern German cohort. Brain Res..

[13] Romano S., Savva G.M., Bedarf J.R., Charles I.G., Hildebrand F., Narbad A. (2021). Meta-analysis of the Parkinson’s disease gut microbiome suggests alterations linked to intestinal inflammation. NPJ Parkinson’s Dis..

[14] Fong W., Li Q., Yu J. (2020). Gut microbiota modulation: A novel strategy for prevention and treatment of colorectal cancer. Oncogene.

[15] Ramirez J., Guarner F., Bustos Fernandez L., Maruy A., Sdepanian V.L., Cohen H. (2020). Antibiotics as Major Disruptors of Gut Microbiota. Front. Cell. Infect. Microbiol..

[16] Gillis J.C., Brogden R.N. (1995). Rifaximin. A review of its antibacterial activity, pharmacokinetic properties and therapeutic potential in conditions mediated by gastrointestinal bacteria. Drugs.

[17] Taylor D.N., Hamer D.H., Shlim D.R. (2017). Medications for the prevention and treatment of travellers’ diarrhea. J. Travel Med..

[18] Caraceni P., Vargas V., Solà E., Alessandria C., de Wit K., Trebicka J., Angeli P., Mookerjee R.P., Durand F., Pose E. (2021). The Use of Rifaximin in Patients With Cirrhosis. Hepatology.

[19] Ponziani F.R., Scaldaferri F., Petito V., Paroni Sterbini F., Pecere S., Lopetuso L.R., Palladini A., Gerardi V., Masucci L., Pompili M. (2016). The Role of Antibiotics in Gut Microbiota Modulation: The Eubiotic Effects of Rifaximin. Dig. Dis..

[20] Fasano A., Bove F., Gabrielli M., Petracca M., Zocco M.A., Ragazzoni E., Barbaro F., Piano C., Fortuna S., Tortora A. (2013). The role of small intestinal bacterial overgrowth in Parkinson’s disease. Mov. Disord..

[21] Suhocki P.V., Ronald J.S., Diehl A.M.E., Murdoch D.M., Doraiswamy P.M. (2022). Probing gut-brain links in Alzheimer’s disease with rifaximin. Alzheimer’s Dement. Transl. Res. Clin. Interv..

[22] Ekstrand M.I., Galter D. (2009). The MitoPark Mouse—An animal model of Parkinson’s disease with impaired respiratory chain function in dopamine neurons. Parkinsonism Relat. Disord..

[23] Schneider C.A., Rasband W.S., Eliceiri K.W. (2012). NIH Image to ImageJ: 25 years of image analysis. Nat. Methods.

[24] Young K., Morrison H. (2018). Quantifying Microglia Morphology from Photomicrographs of Immunohistochemistry Prepared Tissue Using ImageJ. J. Vis. Exp..

[25] Obrenovich M.E.M. (2018). Leaky Gut, Leaky Brain?. Microorganisms.

[26] Lasek-Bal A., Kokot A., Gendosz de Carrillo D., Student S., Pawletko K., Krzan A., Puz P., Bal W., Jędrzejowska-Szypułka H. (2020). Plasma Levels of Occludin and Claudin-5 in Acute Stroke Are Correlated with the Type and Location of Stroke but Not with the Neurological State of Patients-Preliminary Data. Brain Sci..

[27] Jiao X., He P., Li Y., Fan Z., Si M., Xie Q., Chang X., Huang D. (2015). The Role of Circulating Tight Junction Proteins in Evaluating Blood Brain Barrier Disruption following Intracranial Hemorrhage. Dis. Markers.

[28] Carabotti M., Scirocco A., Maselli M.A., Severi C. (2015). The gut-brain axis: Interactions between enteric microbiota, central and enteric nervous systems. Ann. Gastroenterol..

[29] Mogilevski T. (2021). The bi-directional role of the gut-brain axis in inflammatory and other gastrointestinal diseases. Curr. Opin. Gastroenterol..

[30] Valdes A.M., Walter J., Segal E., Spector T.D. (2018). Role of the gut microbiota in nutrition and health. BMJ.

[31] Cryan J.F., O’Riordan K.J., Sandhu K., Peterson V., Dinan T.G. (2020). The gut microbiome in neurological disorders. Lancet Neurol..

[32] Cirstea M.S., Yu A.C., Golz E., Sundvick K., Kliger D., Radisavljevic N., Foulger L.H., Mackenzie M., Huan T., Finlay B.B. (2020). Microbiota Composition and Metabolism Are Associated With Gut Function in Parkinson’s Disease. Mov. Disord..

[33] Petrov V.A., Saltykova I.V., Zhukova I.A., Alifirova V.M., Zhukova N.G., Dorofeeva Y.B., Tyakht A.V., Kovarsky B.A., Alekseev D.G., Kostryukova E.S. (2017). Analysis of Gut Microbiota in Patients with Parkinson’s Disease. Bull. Exp. Biol. Med..

[34] Scheperjans F., Aho V., Pereira P.A., Koskinen K., Paulin L., Pekkonen E., Haapaniemi E., Kaakkola S., Eerola-Rautio J., Pohja M. (2015). Gut microbiota are related to Parkinson’s disease and clinical phenotype. Mov. Disord..

[35] Yu X., Jin Y., Zhou W., Xiao T., Wu Z., Su J., Gao H., Shen P., Zheng B., Luo Q. (2022). Rifaximin Modulates the Gut Microbiota to Prevent Hepatic Encephalopathy in Liver Cirrhosis Without Impacting the Resistome. Front. Cell. Infect. Microbiol..

[36] Jørgensen S.F., Macpherson M.E., Bjørnetrø T., Holm K., Kummen M., Rashidi A., Michelsen A.E., Lekva T., Halvorsen B., Trøseid M. (2019). Rifaximin alters gut microbiota profile, but does not affect systemic inflammation—A randomized controlled trial in common variable immunodeficiency. Sci. Rep..

[37] Bajaj J.S., Heuman D.M., Sanyal A.J., Hylemon P.B., Sterling R.K., Stravitz R.T., Fuchs M., Ridlon J.M., Daita K., Monteith P. (2013). Modulation of the metabiome by rifaximin in patients with cirrhosis and minimal hepatic encephalopathy. PLoS ONE.

[38] Kuti D., Winkler Z., Horváth K., Juhász B., Paholcsek M., Stágel A., Gulyás G., Czeglédi L., Ferenczi S., Kovács K.J. (2020). Gastrointestinal (non-systemic) antibiotic rifaximin differentially affects chronic stress-induced changes in colon microbiome and gut permeability without effect on behavior. Brain Behav. Immun..

[39] Sinkala E., Zyambo K., Besa E., Kaonga P., Nsokolo B., Kayamba V., Vinikoor M., Zulu R., Bwalya M., Foster G.R. (2018). Rifaximin Reduces Markers of Inflammation and Bacterial 16S rRNA in Zambian Adults with Hepatosplenic Schistosomiasis: A Randomized Control Trial. Am. J. Trop. Med. Hyg..

[40] Larsen J.M. (2017). The immune response to Prevotella bacteria in chronic inflammatory disease. Immunology.

[41] Iljazovic A., Roy U., Gálvez E.J.C., Lesker T.R., Zhao B., Gronow A., Amend L., Will S.E., Hofmann J.D., Pils M.C. (2021). Perturbation of the gut microbiome by Prevotella spp. enhances host susceptibility to mucosal inflammation. Mucosal Immunol..

[42] Kumar P.S., Griffen A.L., Barton J.A., Paster B.J., Moeschberger M.L., Leys E.J. (2003). New bacterial species associated with chronic periodontitis. J. Dent. Res..

[43] Lucke K., Miehlke S., Jacobs E., Schuppler M. (2006). Prevalence of Bacteroides and Prevotella spp. in ulcerative colitis. J. Med. Microbiol..

[44] Elinav E., Strowig T., Kau A.L., Henao-Mejia J., Thaiss C.A., Booth C.J., Peaper D.R., Bertin J., Eisenbarth S.C., Gordon J.I. (2011). NLRP6 inflammasome regulates colonic microbial ecology and risk for colitis. Cell.

[45] Brown E.M., Ke X., Hitchcock D., Jeanfavre S., Avila-Pacheco J., Nakata T., Arthur T.D., Fornelos N., Heim C., Franzosa E.A. (2019). Bacteroides-Derived Sphingolipids Are Critical for Maintaining Intestinal Homeostasis and Symbiosis. Cell Host Microbe.

[46] Mazmanian S.K., Round J.L., Kasper D.L. (2008). A microbial symbiosis factor prevents intestinal inflammatory disease. Nature.

[47] Parada Venegas D., De la Fuente M.K., Landskron G., González M.J., Quera R., Dijkstra G., Harmsen H.J.M., Faber K.N., Hermoso M.A. (2019). Short Chain Fatty Acids (SCFAs)-Mediated Gut Epithelial and Immune Regulation and Its Relevance for Inflammatory Bowel Diseases. Front. Immunol..

[48] Sibai M., Altuntaş E., Yıldırım B., Öztürk G., Yıldırım S., Demircan T. (2020). Microbiome and Longevity: High Abundance of Longevity-Linked Muribaculaceae in the Gut of the Long-Living Rodent Spalax leucodon. OMICS.

[49] Baldini F., Hertel J., Sandt E., Thinnes C.C., Neuberger-Castillo L., Pavelka L., Betsou F., Krüger R., Thiele I., Aguayo G. (2020). Parkinson’s disease-associated alterations of the gut microbiome predict disease-relevant changes in metabolic functions. BMC Biology.

[50] Mao L., Zhang Y., Tian J., Sang M., Zhang G., Zhou Y., Wang P. (2021). Cross-Sectional Study on the Gut Microbiome of Parkinson’s Disease Patients in Central China. Front. Microbiol..

[51] Ferrari C.C., Tarelli R. (2011). Parkinson’s disease and systemic inflammation. Parkinsons Dis..

[52] Brudek T. (2019). Inflammatory Bowel Diseases and Parkinson’s Disease. J. Parkinsons Dis..

[53] Weimers P., Halfvarson J., Sachs M.C., Saunders-Pullman R., Ludvigsson J.F., Peter I., Burisch J., Olén O. (2018). Inflammatory Bowel Disease and Parkinson’s Disease: A Nationwide Swedish Cohort Study. Inflamm. Bowel Dis..

[54] Bower J.H., Maraganore D.M., Peterson B.J., Ahlskog J.E., Rocca W.A. (2006). Immunologic diseases, anti-inflammatory drugs, and Parkinson disease: A case-control study. Neurology.

[55] Samii A., Etminan M., Wiens M.O., Jafari S. (2009). NSAID use and the risk of Parkinson’s disease: Systematic review and meta-analysis of observational studies. Drugs Aging.

[56] Kinashi Y., Hase K. (2021). Partners in Leaky Gut Syndrome: Intestinal Dysbiosis and Autoimmunity. Front. Immunol..

[57] Chan L., Chung C.-C., Chen J.-H., Yu R.-C., Hong C.-T. (2021). Cytokine Profile in Plasma Extracellular Vesicles of Parkinson’s Disease and the Association with Cognitive Function. Cells.

[58] Qin X.Y., Zhang S.P., Cao C., Loh Y.P., Cheng Y. (2016). Aberrations in Peripheral Inflammatory Cytokine Levels in Parkinson Disease: A Systematic Review and Meta-analysis. JAMA Neurol..

[59] Reale M., Iarlori C., Thomas A., Gambi D., Perfetti B., Di Nicola M., Onofrj M. (2009). Peripheral cytokines profile in Parkinson’s disease. Brain Behav. Immun..

[60] Leclercq S., Mian F.M., Stanisz A.M., Bindels L.B., Cambier E., Ben-Amram H., Koren O., Forsythe P., Bienenstock J. (2017). Low-dose penicillin in early life induces long-term changes in murine gut microbiota, brain cytokines and behavior. Nat. Commun..

[61] Logsdon A.F., Erickson M.A., Rhea E.M., Salameh T.S., Banks W.A. (2018). Gut reactions: How the blood-brain barrier connects the microbiome and the brain. Exp. Biol. Med..

[62] Umemura A., Oeda T., Yamamoto K., Tomita S., Kohsaka M., Park K., Sugiyama H., Sawada H. (2015). Baseline Plasma C-Reactive Protein Concentrations and Motor Prognosis in Parkinson Disease. PLoS ONE.

[63] Martin-Ruiz C., Williams-Gray C.H., Yarnall A.J., Boucher J.J., Lawson R.A., Wijeyekoon R.S., Barker R.A., Kolenda C., Parker C., Burn D.J. (2020). Senescence and Inflammatory Markers for Predicting Clinical Progression in Parkinson’s Disease: The ICICLE-PD Study. J. Parkinsons Dis..

[64] Koo H.L., DuPont H.L. (2010). Rifaximin: A unique gastrointestinal-selective antibiotic for enteric diseases. Curr. Opin. Gastroenterol..

[65] Bass N.M., Mullen K.D., Sanyal A., Poordad F., Neff G., Leevy C.B., Sigal S., Sheikh M.Y., Beavers K., Frederick T. (2010). Rifaximin treatment in hepatic encephalopathy. N. Engl. J. Med..

[66] Amboni M., Barone P., Hausdorff J.M. (2013). Cognitive contributions to gait and falls: Evidence and implications. Mov. Disord. Off. J. Mov. Disord. Soc..

[67] Howard C.E., Chen C.L., Tabachnik T., Hormigo R., Ramdya P., Mann R.S. (2019). Serotonergic Modulation of Walking in Drosophila. Curr. Biol..

