# Supplementary material for: Rifaximin Modifies Gut Microbiota and Attenuates Inflammation in Parkinson’s Disease: Preclinical and Clinical Studies
Source: Cells. 2022 Nov 2;11(21):3468. doi: 10.3390/cells11213468 (PMC9656351; doi:10.3390/cells11213468)

Supplementary Figure

Supplementary Figure 1. The full blot of MitoPark serum cytokine analysis

Figure 1A Interleukin (IL)-1β

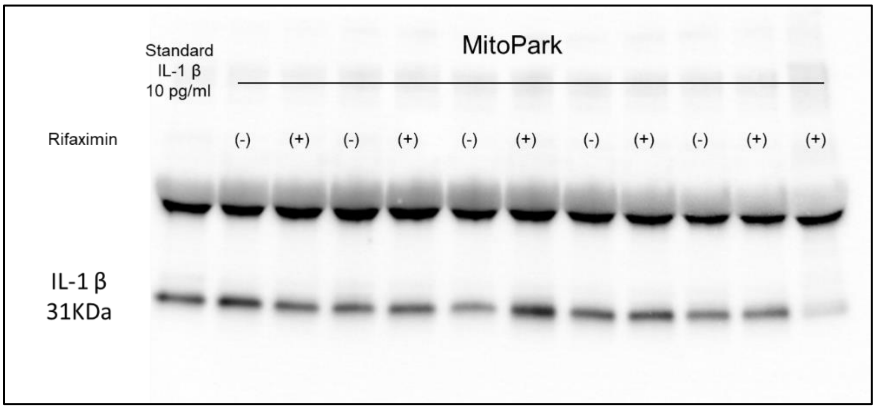

Figure 1B IL-6

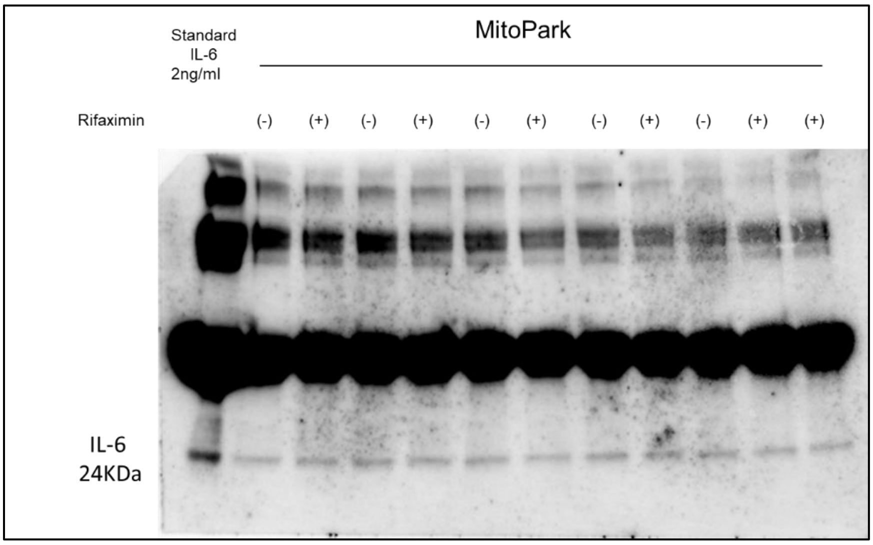

Figure 1C Tumor necrosis factor (TNF)-1α

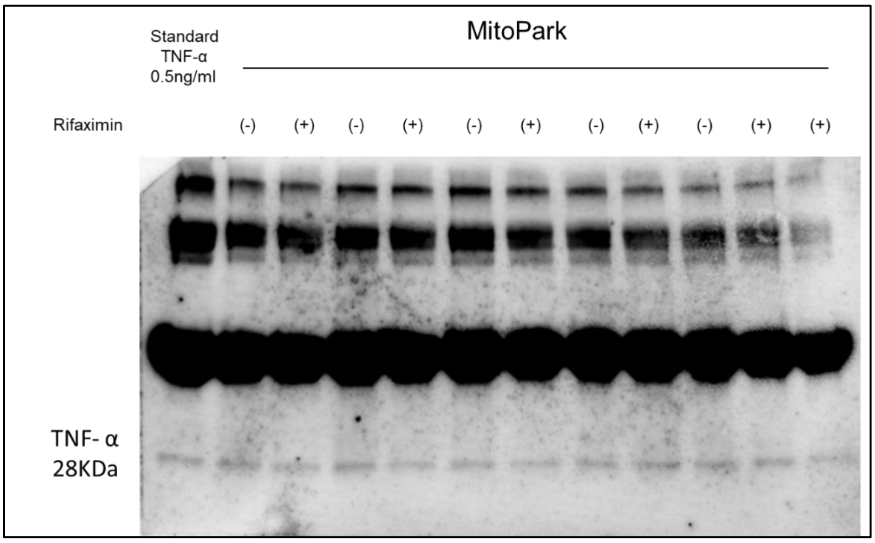

Supplementary Figure 2. The representative western blot analysis for the assessment of the ratio of the expression of arginase-1 (a marker of M2 microglia) to CD86 (a marker of M1 microglia) in the midbrain substantia nigra (SNR) and hippocampus.

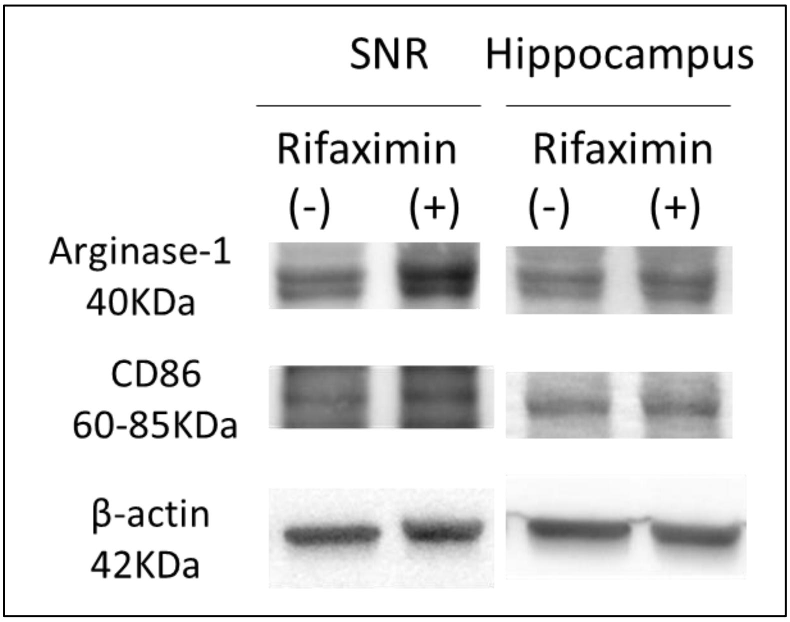

Supplementary Figure 3. The difference in the relative abundance of major bacterial gena in the study participants before and 6 months after 7-day rifaximin treatment. Data are presented as medians with the first and third quartiles. None of the listed results reached statistic significant.

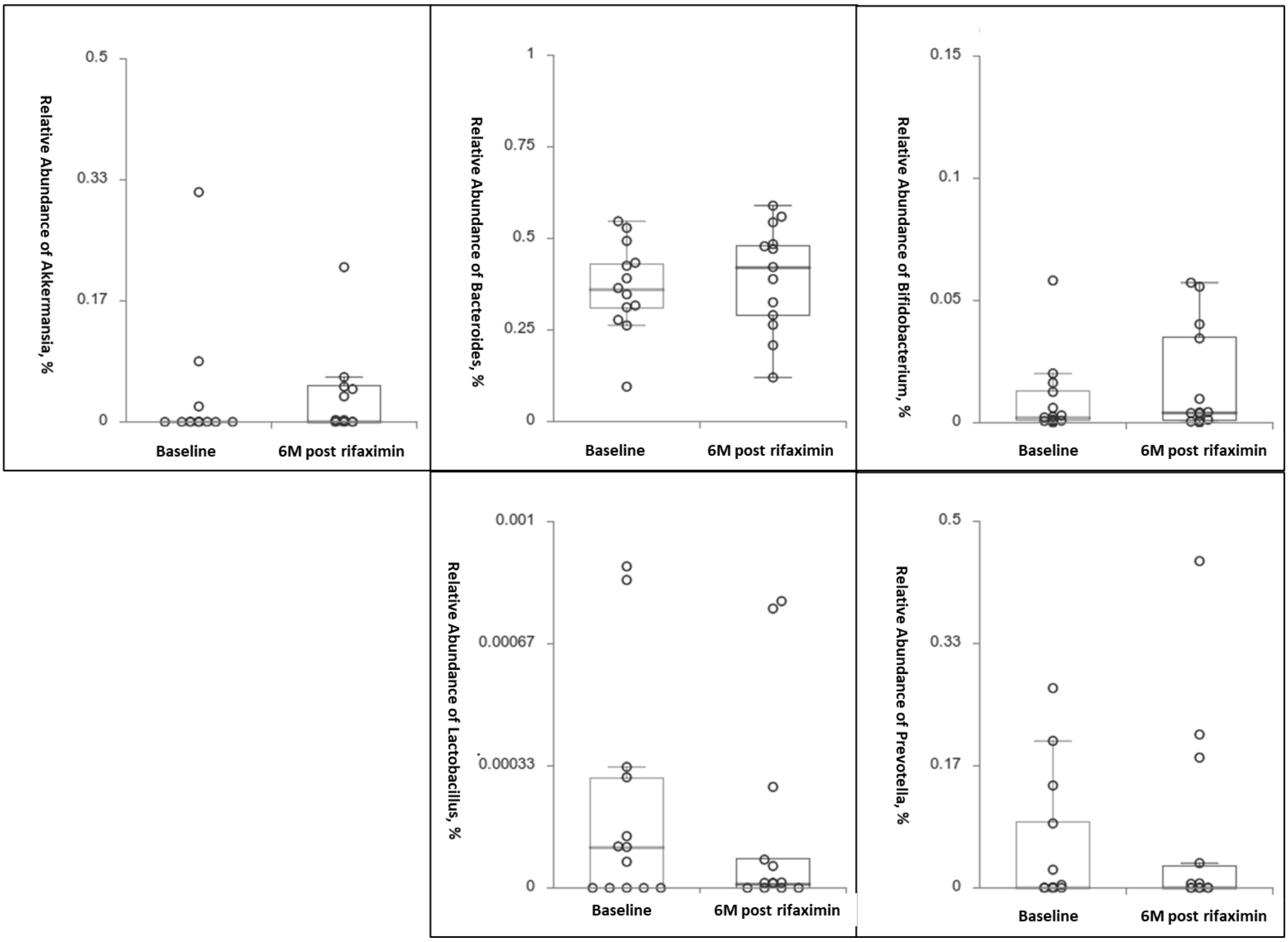

Supplement: Supplementary file 1 [file cells-11-03468-s001.zip › cells-1892984-supplementary.pdf]
